# Dataset of replicate Apollo sample magnetizations bearing on impacts and absence of a long-lived lunar dynamo

**DOI:** 10.1038/s41597-024-03626-0

**Published:** 2024-07-20

**Authors:** Rory D. Cottrell, Tinghong Zhou, John A. Tarduno

**Affiliations:** 1 Department of Earth and Environmental Sciences, University of Rochester, Rochester, N.Y. 14627 USA; 2 Department of Physics and Astronomy, University of Rochester, Rochester, N.Y. 14627 USA; 3 Laboratory for Laser Energetics, University of Rochester, Rochester, NY 14623 USA

**Keywords:** Core processes, Rings and moons, Core processes, Magnetospheric physics, Early solar system

## Abstract

The absence or presence of a lunar paleomagnetosphere is important because it bears directly on the volatile content of the regolith and exploration targets for Artemis and other missions to the Moon. Recent paleointensity study of samples from the Apollo missions has readdressed this question. Multiple specimens from a young 2-million-year-old glass shows a strong magnetization compatible with that induced by charge-separation in an impact plasma, whereas paleointensities of single crystals yield evidence for null magnetizations spanning 3.9 to 3.2 Ga. Together, these data are consistent with an impact mechanism for the magnetization of some lunar samples, and absence of a long-lived lunar core dynamo and paleomagnetosphere recorded in other samples. Here, we present a dataset that allows researchers to examine replicates of these measurements. For the glass, we present data from specimens that fail standard paleointensity selection criteria but nevertheless imply a complex, changing magnetic field environment. For the single crystals, the replicate measurements further illustrate the initial zero magnetization state of these materials.

## Background & Summary

A reinvestigation of the lunar Apollo record has recently offered a new paradigm whereby the Moon lacked a magnetic field for most of its history, at least since 3.9 billion years ago^1^, superseding prior interpretations of a paradoxically strong and long-lived lunar core dynamo^2^. Understanding the past lunar magnetic environment is of topical importance as we are on the eve of renewed scientific exploration of the Moon by the Artemis and other missions. Without a past core dynamo, solar winds would not be blocked by a paleomagnetosphere, and a greater volatile content in the lunar regolith is expected^3^. Moreover, the lack of magnetic shielding suggests components of Earth’s ancient atmosphere could be transported through Earth’s paleomagnetotail^4,5^ to the lunar surface, similar to processes occurring today^6^. This raises the possibility that otherwise inaccessible records of Earth’s ancient atmosphere might be preserved in buried lunar soils^7^, representing a profound target for future exploration.

The new paradigm was motivated by the observation of strong paleofield strengths recovered by thermal paleointensity analyses of Apollo 64455, a 2 million-year-old glass formed from the impact recorded by South Ray crater^8^. The Moon currently lacks a core dynamo and there is no reason to believe it had one 2 million years ago because its thermal state would not have been substantially different from that of today. These 64455 field strengths^1^ match those predicted by the charge separation mechanism, a process documented in laboratory and modeling studies associated with asteroid and comet impacts^9–11^. This further implies that the 64455 specimens yielding relatively strong paleointensities were quenched on second timescales near the rim of South Ray crater^1^. Based on comparisons with experimental analogs, coolings rates of 8 °C s^−1^ have been reported for 64455, but these analyses best detected minimum rates^12^. See *et al*.^13^ inferred quenching on second timescales compatible with the presence of an impact plasma magnetic field at South Ray crater^1^. Thus, impact magnetization thus provides one mechanism to account for prior high anomalous magnetizations^2^ from lunar samples.

In paleointensity studies, it is typical to establish selection criteria. These reflect an analysis that excludes data reflecting nonideal magnetic recording and/or if the magnetic minerals have changed during the analysis. In the thermal studies^1^ of Apollo 64455 (Methods), the success rate was 12%, a rate comparable to investigations of terrestrial materials^14,15^. Displaying data from unsuccessful paleointensity experiments can can exceed commonplace journal figure limits, (cf. ref. ^1^), and such data are not typically contributed to databases. But in this case, these data can provide further information on the nature of the past field. In particular, while not meeting one criterion (or more) to establish a robust paleointensity value, they may be sufficient to establish the presence of a field. In addition, if the ambient field is changing during the time of magnetization - unusual for rocks but possible if not likely for glasses within an impact plasma^1^ - the sample could hold valuable information on that process. For these reasons, we present such data on Apollo 64455 specimens previously rejected following selection criteria (Methods). Reasons for rejection are varied, and as reported in Tarduno *et al*.^1^ include “evidence for multiple components and/or changing directions after field-off thermal treatments. Others show evidence for thermally induced chemical or structural changes and/or nonideal recording behavior." As will be discussed below, while failing to meet paleointensity selection criteria, some of these data may provide addition information on the nature of the ambient field environment.

The discovery of the magnetization of Apollo 64455 also prompted a reanalysis of the high paleointensities produced in some studies (but already challenged in others^16^). A particular point of contention has been the use of nonthermal techniques on lunar samples to obtain paleointensity values. If faithfully recording a past dynamo, lunar rocks should have magnetic minerals recording a thermoremanent magnetization acquired during cooling. In the case of 64455, these carriers have been documented by scanning electron microscope analyses of a diverse assemblage of FeNi inclusions^1^. The gold standard to recover any paleointensity in this case is a thermal (Thellier) measurement^17^. However, lunar magnetic minerals are especially prone to alteration with heating, due to their reduced state^18^ and propensity for structural change^17^. To avoid such alteration, many workers have used nonthermal methods involving alternating field demagnetization and the application of isothermal remanent magnetizations^2,19^. Irrespective of their widespread use, the accuracy of these methods remains highly contentious^1,16,17^. Another outstanding issue with the prior Apollo data regards magnetic domain state. Single domain, or single domain-like (single vortex or pseudosingle domain) magnetic carriers are needed for individual robust paleointensity determinations. Only these domain states satisfy Thelliers’ recording laws^17^, but lunar whole rock samples are typically dominated by large multidomain (MD) grains, which can be expected to have highly complex behavior based on the distribution of defects and domain walls^17^. Low temperature treatments, sometimes used to remove MD magnetizations in terrestrial samples, would not be advisable given the complex behavior between domain walls and twinning^17,20^.

To address these challenges, the single crystal paleointensity technique (SCP)^21,22^ was applied to samples from five Apollo samples ranging in age from 3.2 to 3.9 Ga. The SCP technique isolates single silicate grains that contain minute magnetic minerals, excluding MD grains. In addition, rapid CO_2_ laser heating^21,23^ was used to limit alteration; alteration checks were used to assess the effectiveness of this approach (Methods). Data from these checks are inconsistent with alteration accompanying heating being significant (including during the first thermal step). Alteration is a function of the magnetic mineral inclusion and host silicate chemistry and heating time, and heating in the presence of a field is a very sensitive test. If alteration had resulted in the formation of new magnetic minerals, apparent recording efficiencies ≫ 100% should have be observed (but these were not), and alteration checks should fail. The lack of evidence for alteration the lunar SCP analyses, relative to the 64455 results, can be attributed to the limited number of total steps. However, it is also possible that the FeNi carriers in the 64455 glass are more susceptible to chemical and structual change^24^ with heating.

The natural remanent magnetization (NRM) of a sample was typically measured multiple times, as was the magnetization after thermal demagnetization to 590 °C, and after the alteration check following the heating in the presence of a 20 *μ*T field. The NRM data show some consistency, whereas the data after heating in zero field at 590 °C do not, consistent with the zero magnetization state. The first measurements of NRM, 590 °C demagnetization, and 590 °C demagnetization after the heating in a 20 *μ*T field, were plotted in ref. ^1^. and reported in the dataset accompanying that paper. For completeness, here we report the replicate measurements.

Although the unstable magnetic directions are consistent with null ambient fields on the Moon during the cooling of the magnetic minerals in the crystals, detecting a zero magnetization is ultimately dependent on magnetometer sensitivity. The first measurement collected after initial demagnetization at 590 °C, and after heating to 590 °C, and cooling in a 20 *μ*T field, were used to calculate nominal paleointensities for each crystal, and reported in Tarduno *et al*.^1^. These values are nominal, and should not be used as a bound on paleointensity, because as highlighted in Tarduno *et al*.^1^, one of the values used (i.e., the magnetization after initial demagnetization at 590 °C) is unstable. Instead, a true bound on the maximum paleointensity allowable by the data can be calculated using the minimum magnetization strength detectable by the ultrasensitive magnetometer employed in the study. These values are presented below. While there is no evidence for ambient lunar fields in the 590 °C data as strong as these values, they nevertheless define a baseline of ~0.2 to ~0.5 *μ*T below which fields cannot be evaluated with the present data.

### Glass: Apollo 64455,24

Thermal paleointensity data can be displayed in standard NRM lost versus TRM gained (Arai) plots based on the Thellier-Thellier method, as modified by Coe *et al*.^25^ to allow for repeated partial thermal magnetization (pTRM) alteration checks. For some experiments, an AF pre-treatment was used prior to Thellier-Thellier paired heating steps to test for and remove spurious fields.

**Fig. 1:**
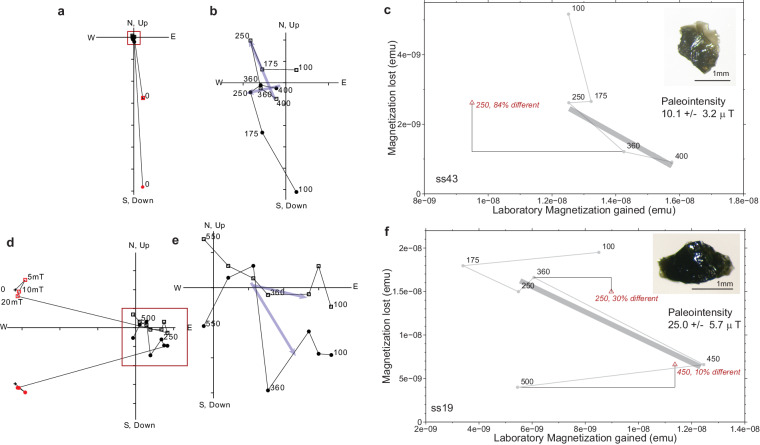
Apollo 64455 glass subsamples that did not pass reliability criteria but showed hints of linearity in orthogonal vector plots. (**a,d**) Full orthogonal vector plots for subsamples ss43 and ss19, respectively. Open squares, vertical projection of magnetization directions. Closed circles, horizontal projection of magnetization directions. AF pre-treatment steps are as red symbols. (**b,e**) Enlargement of origin of orthogonal vector plots. Labeled steps are in °C. Blue arrows indicate potential linear magnetization directions. (**c,e**) Natural remanent magnetization lost plotted against thermal remanent magnetization gained for subsamples ss43 and ss19 respectively. Grey circles represent paired heating steps, red triangles represent partial thermal remanent magnetization checks. The thick gray line represents a nominal range of data that could be fit for paleointensity estimates. The absolute value of the slope of the best fit line is multiplied by the laboratory field value used (18 *μ*T). Inset: photomicrograph of the subsample with 1 mm scale bar. Data can be found in the figshare repository^26^.

**Fig. 2:**
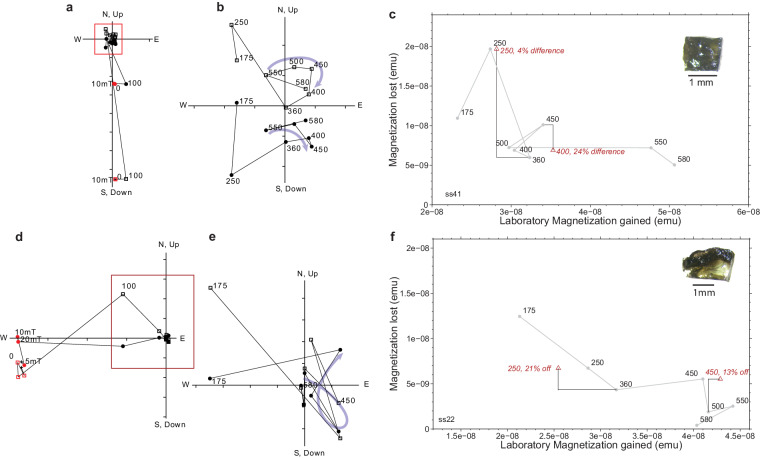
Apollo 64455 glass subsamples that did not pass reliability criteria displaying a changing ambient magnetic field. (**a,d**) Full orthogonal vector plots for subsamples ss41 and ss22, respectively. Open squares, vertical projection of magnetization directions. Closed circles, horizontal projection of magnetization directions. AF pre-treatment steps are as red symbols. (**b,e**) Enlargement of origin of orthogonal vector plots. Labeled steps are in °C. Blue arrows indicate changing magnetization directions over a temperature interval. (**c,e**) Natural remanent magnetization lost plotted against thermal remanent magnetization gained for subsamples ss41 and ss22 respectively. Grey circles represent paired heating steps, red triangles represent partial thermal remanent magnetization checks. Inset: photomicrograph of the subsample with 1 mm scale bar. Data can be found in the figshare repository^26^.

Examination of field off directional data in orthogonal vector plots can be particularly revealing. For example, some of these data reveal linear directional components, and nominal paleointensities can be calculated (Fig. 1). Other specimens show a changing vector, consistent with a changing ambient field (Fig. 2). The directions, as viewed on orthogonal vector plots, are not random and imply the presence of an ambient field. Plots for all the data not meeting paleointensity criteria^1^ (n=22) are provided^26^ with photomicrographs of specimens that were measured.

### Single crystal measurements: Apollo 14053,262; 12021,30; 12053,283; 12040,209; 71055,2

Twelve crystals from these samples yielded evidence for null magnetizations. However, determining a zero magnetization is itself challenging, and several replicate measurements were taken to confirm the null magnetization state.

**Fig. 3:**
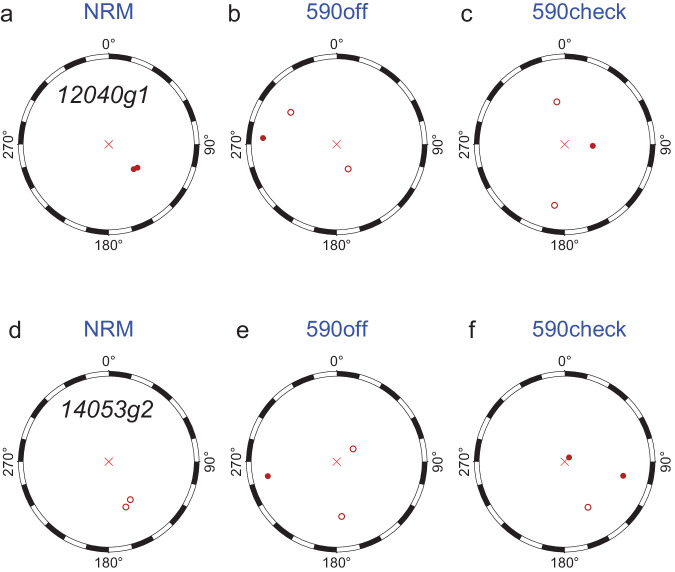
Stereonets of field-off steps (0.0, 590.0, 590.3) for Apollo single silicate measurements as reported in Tarduno *et al*.^1^ for samples 12040g1 and 14053g2. Closed symbols, directions that plot on the lower hemisphere; open symbols, directions that plot on the upper hemisphere. Data can be found in the figshare repository^26^.

Replicate NRM measurements and nominal magnetization directions after CO_2_ laser demagnetization at 590 °C can be displayed on stereonets as shown in Fig. 3 for samples 12041g1 and 14053g2 (sample naming convention from Tarduno *et al*.^1^). These show that for most of the crystals there is consistency of the NRM direction (Fig. 3a,d), but this consistency is not seen after demagnetization at 590 °C (Fig. 3b,e). The directions are also inconsistent after the alteration check at 590 °C (Fig. 3c,f).

One crystal (14053g1) was measured after heating to an intermediate temperature (400 °C) during initial tests, and found to be stable. At this relatively low temperature we cannot be assured that the remanence is dominated by single-domain behavior (versus a magnetization held by large pseudosingle or large single domain grains), especially given the slightly relaxed crystal selection criteria which allowed some visible inclusion in the crystals measured^1^. It is possible that the remanance in this crystal observed after 400 °C, but removed after treatment at 590 °C, is a shock remanent magnetization, as has been discussed for magnetization of 14053 bulk samples^27^.

As noted above, while the unstable directions after demagnetization at 590 °C are consistent with null ambient fields on the Moon, the determination of zero is itself limited by magnetometer sensitivity. Considering a magnetometer threshold value (see Methods) the maximum field that cannot be excluded by the data can be calculated from the data for each crystal. These values are presented in Table 1. See Technical Validation for an example of these calculations. We reemphasize that there is no evidence for these fields in the 590 °C data. Instead, they are a baseline below which fields cannot be evaluated with the present data.

**Table 1:** Magnetization Thresholds.

Sample	M_590_ 20 *μ*T^†^	Max intensity^‡^
	emu	*μT*
12021g2	7.011E-09	0.23
12021g3	6.292E-09	0.26
12040g1	8.002E-09	0.20
12040g2	6.021E-09	0.27
12040g3	6.292E-09	0.26
12053g1	5.283E-09	0.31
12053g2	7.982E-09	0.20
14053g1	3.203E-09	0.51
14053g2	5.294E-09	0.31
14053g3	4.523E-09	0.36
71055g1	3.883E-09	0.42
71055g3	4.202E-09	0.39

^†^Magnetization acquired after heating to 590 °C in presence of a 20 *μ*T field.

^‡^Magnetization intensity threshold (value below which a magnetic intensity cannot be detected with the available data).

## Methods

Methods follow those described in Tarduno *et al*.^1^ and are summarized below. Apollo samples 64455,24; 14053,262; 12021,30; 12053,283; 12040,209; and 71055,2 were collected by Kristin Lawrence at the Johnson Space Laboratory and carried to the University of Rochester for analysis. The 64455 glass was attached to the rock substrate and easily separated by hand. The glass was further crushed using nonmagnetic tools to mm and less sizes for rock magnetic and paleomagnetic analyses^1^. Similarly, rock samples of the Apollo rocks were gently crushed using nonmagnetic tools to isolate individual feldspar or pyroxene crystals.

Initial experiments conducted in 2014 and 2015 on glass subsamples from 64455 (n=13) did not utilize an AF pretreatment prior to thermal demagnetization. One successful Thellier sample reported in Tarduno *et al*.^1^ is from this group. Experiments after this time (n = 12) included AF demagnetization along 6 directions (paired positive and negative demagnetizations along sample X, Y and Z axes) at steps of 5 and 10 mT. Some samples had additional steps of 2.5, 7.5 and 20 mT. For nearly all samples, AF cleaning did not significantly change the NRM direction or magnitude. Two successful Thellier samples reported in Tarduno *et al*.^1^ are in this group.

### Magnetometer measurements

Magnetic measurements were made with the ultrasensitive William S. Goree Inc. (WSGI) three-component DC SQUID magnetometer (6.3 mm room temperature access bore) located in the magnetically shielded room at the University of Rochester. The ambient magnetic field within the room is <200 nT. Stable remanences as low as ~4 × 10^−11^ emu (~4 × 10^−14^ A m^2^) have been recorded using this magnetometer. Here, we use a sensitivity threshold of 8 × 10^−11^ emu (8 × 10^−14^ A m^2^) which is more representative of typical measurements.

### Thermal measurements of glass and single silicate samples

Glass samples were mounted in 2 mm fused quartz boxes using nonmagnetic adhesives whose purity has been confirmed through comparative studies between the University of Rochester and AIST, Japan, using a scanning SQUID microscope^28^. These boxes were placed on quartz rods for measurement.

Samples were heated using a Synrad 20 CO_2_ laser for Thellier-Coe paleointensity analyses of 64455 glass. Heating times were either 90 seconds or 120 seconds depending on the specimen size (<1 mm or 1–2 mm, respectively). Heating times were the same for thermal analyses of single silicate crystals, but the very weak natural remanent magnetizations and null readings after heating to 590 °C necessitated a different procedure^1^, described below.

For paleointensity determinations, samples are typically treated in paired heating steps and allowed to cool in a zero magnetic field, or in the presence of a known laboratory field. The loss of the sample’s natural remanent magnetization (NRM) is plotted against the acquisition of the laboratory-induced thermal remanent magnetization (TRM). The TRM is the calculated vector subtraction of field-on and field-off steps. Alteration checks can be done in two ways. A repeat measurement can be done at lower temperatures to replicate the acquisition of the laboratory field (a partial TRM, pTRM check, as applied for 64455 specimens). A repeated measurement in a zero magnetic field after heating in the presence of a lab field at the same temperature can also be used to gauge alteration (as applied for the single silicate crystals^1^). Magnetization steps for Apollo 64455 are designated as field-off, field-on, and pTRM check (field-on), with the following labels:

- *#*.0 for field-off steps
- *#*.1 for field-on steps
- *#*.2 for pTRM checks (field-on)

where *#* is the demagnetization step.

The NRMs of individual crystals were measured to find specimens with moments of approximately 1 × 10^−9^ emu or greater. For these samples, a slightly different 4-step procedure was used^1^: 1. the crystal was heated to 590 °C in a zero field and measured (*#*.0); 2. the sample was reheated to 590 °C in the presence of a 20 *μ*T applied field and measured (*#*.1); 3. the crystal was heated to 590 °C in a zero field and measured (alteration check, *#*.3); and 4. the crystal was heated to 590 °C in a 40 *μ*T field and measured (*#*.4).

NRMs of the single silicate crystals were generally consistent in direction space. After heating to 590 °C in a zero field (Steps 1 and 3), magnetization directions were inconsistent, suggesting a null magnetization state. However, a null magnetization measured with a WSGI (or 2G) SQUID magnetometer will still register a value (i.e., a resultant of the 3 measured components), and this nominal magnetization was previously shown in figures and data sets associated with ref. ^1^. To further confirm the null state, replicate measurements were taken for Steps 1 and 3. These data are reported in the dataset featured here^26^.

### Paleointensity Selection criteria

The 64455 Thellier data were analyzed in ref. ^1^ using selection criteria following Cottrell and Tarduno^29^ and modified as follows: NRM loss versus TRM gained data must show a linear relationship (R^2^ ~ 0.9) with 4 or more points defining the slope. These points should be evenly distributed and pTRM checks should be with 15%. For the 64455 data the maximum angular deviation of the line fit defining the magnetization was relaxed, and a three-point sliding window was used for the orthogonal vector plots to reduce noise and identify the characteristic remanent magnetization temperature range.

Reasons for de-selection are provided in Table 2. We note that some specimens did not acquire TRM in a regular fashion, or showed directions that trended out of, rather toward, the origin of their respective orthogonal vector plots. These inconsistencies, which may be a sign of alteration, arguable supersede standard selection criteria^29–31^.

**Table 2:** De-selection criteria.

Sample	n	T Range	MAD	R2	pTRM checks	Failure criteria	Comment
ss19^†▹^	4	175–450	30.1	0.94	250 (30%), 450 (9%)	pTRM, MAD, n	not origin trending
ss43^†▹^	3	250–400	10.0	0.90	250 (85%)	pTRM, n	trend near origin
ss22^‡▹^	4	250–500	35.4	0.38	250 (22%) 450 (13%)	pTRM, MAD, R2	curved
ss41^‡▹^	3	500–580	24.0	0.38	250 (4%), 400 (24%)	pTRM, MAD, R2, n	curved
ss6^∧^	3	175–360	21.0	0.91	—	MAD, R2, n, no pTRM	trending out of origin
ss9^∧^	4	250–550	37.6	0.74	250 (21%)	pTRM, MAD, R2	
ss10^∧^	3	360–550	42.9	0.15	250 (83%)	pTRM, MAD, R2, n	
ss30^∧^	4	175–500	12.5	0.59	450 (10%)	R2	curved
ss34^▹^	5	100–450	12.1	0.06	250 (9%)	R2	trending out of origin
g2^∧^	4^⋆^	100–400^◇^	—	—	—	no fit	no regular TRM acquisition
ss8^∧^	5^⋆^	100–450^◇^	—	—	—	no fit	no regular TRM acquisition
s11^∧^	6^⋆^	100–550^◇^	—	—	250 (22%)	no fit	no regular TRM acquisition
ss18^∧^	5^⋆^	100–450^◇^	—	—	250 (66%)	no fit	no regular TRM acquisition
ss24^∧^	7^⋆^	100–550^◇^	—	—	250 (6%), 450 (42%)	no fit	no regular TRM acquisition
ss27^▹^	6^⋆^	100–550^◇^	—	—	305 (1%), 400 (54%)	no fit	no regular TRM acquisition
ss29^∧^	6^⋆^	100–550^◇^	—	—	450 (25%)	no fit	no regular TRM acquisition
ss36^∧^	4^⋆^	100–450^◇^	—	—	450 (25%)	no fit	trends out of origin
ss38^▹^	4^⋆^*	100–360^◇^	—	—	250 (9%)	no fit	no reg. TRM aquisition; stable direction
ss39^∧^	2^⋆^	100–210^◇^	—	—	—	no fit	trends out of origin
ss45^▹^	4^⋆^	100–360^◇^	—	—	250 (120%)	no fit	no regular TRM acquisition
ss46^▹^	3^⋆^	100–250^◇^	—	—	—	no fit	no regular TRM acquisition
ss48^▹^	2^⋆^	100–175^◇^	—	—	—	no fit	no regular TRM acquisition

Double line separates specimens for which a line fit was not attempted.

n: number of temperature steps used in line fit.

n^⋆^: number of paired field on/field off steps in the experiment.

pTRM (value%): percentage deviant from check value.

^◇^temperature range of paired heating steps.

^∧^sample measured in 2014 and 2015.

^▹^sample measured after 2016.

## Data Records

Replicate measurements, photomicrographs, and additional example plots are stored at Figshare^26^ for Apollo 64455,24 samples reported here. Measurements made with the WSGI 6.3 mm small-bore superconducting SQUID magnetometer for each sample are stored in sheets in Excel files with columns of sample name, demagnetization step, magnetization moment (in electromagnetic units, the units measured with the WSGI magnetometer), declination and inclination. AF demagnetization steps have a ‘mT’ label; all other steps represent degrees Celsius. Plots of orthogonal vector plots, enlargement of the origin of these plots, and diagrams of NRM lost versus TRM gained for 64455 subsamples not shown here are provided as additional PDFs^26^.

Replicate measurements for Apollo samples 14053,262; 12021,30; 12053,283; 12040,209; and 71055,2 are tabulated in separate Excel files following the same format as above. Column names follow the same pattern of sample name, demagnetization step, magnetization moment (in electromagnetic units), declination and inclination. Plots of stereonets of the field off steps as shown in Fig. 3 are provided for samples reported in ref. ^1^ as an additional PDF^26^.

## Technical Validation

PmagPy^30^ was used to look at directional and paleointensity data during data collection. Determination of quality factors as discussed in Tarduno *et al*.^1^ was independently verified using standard paleointensity definitions^31^.

A standalone version of software (PmagPy GUI) can be downloaded (https://github.com/Pmagpy) for multiple platforms. A readme file for its use is provided^26^. All figures were made with Python 3.8 with PyGMT (https://zenodo.org/records/11062720)^32^ wrappers for Generic Mapping Tools^33^.

### Example of threshhold detection limit

A single temperature determination of paleointensity^5^ can be calculated for the single crystal samples through a comparison of the NRM left after heating to 590 °C to the acquisition of TRM in a laboratory field of 20 *μ*T^1^. Magnetic directions after heating to 590 °C are inconsistent, and suggests that any remaining magnetization is at a level below the magnetometer sensitivity, or no remanent magnetization was imparted on the sample at formation^1^. We can compare the acquisition of a laboratory induced magnetic field to the magnetometer sensitivity value (8 × 10^−14^ A m^2^) to calculate a threshold field detection limit.

For 14053g1:

Threshold field detection = Magnetometer sensitivity (8 × 10^−14^ A m^2^) / ${{\rm{TRM}}}_{59{0}^{o}}$ (8 × 10^−12^ A m^2^) × Lab Field (20 *μ*T) = 0.2 *μ*T.

## Data Availability

No custom code was used to generate or process the data described in this manuscript.
